# Simultaneous Quantification of Bioactive Triterpene Saponins Calenduloside E and Chikusetsusaponin IVa in Different Plant Parts of Ten Amaranthaceae Species by UPLC-ESI-MS/MS Method

**DOI:** 10.3390/molecules30051088

**Published:** 2025-02-27

**Authors:** Karolina Grabowska, Paweł Żmudzki, Agnieszka Galanty, Irma Podolak

**Affiliations:** 1 Department of Pharmacognosy, Jagiellonian University Medical College, 9 Medyczna Str., 30-688 Cracow, Poland; karolina1.grabowska@uj.edu.pl (K.G.); irma.podolak@uj.edu.pl (I.P.); 2 Department of Medicinal Chemistry, Jagiellonian University Medical College, 9 Medyczna Str., 30-688 Cracow, Poland; pawel.zmudzki@uj.edu.pl; 3 Center for the Development of Therapies for Civilization and Age-Related Diseases, Jagiellonian University Medical College, Skawińska 8, 31-066 Krakow, Poland

**Keywords:** calenduloside E, chikusetsusaponin IVa, UPLC-MS/MS, quantification, triterpene saponins, Amaranthaceae, *Chenopodium*, *Atriplex*, *Oxybasis*, *Blitum*

## Abstract

Calenduloside E (CE) and chikusetsusaponin IVa (ChIVa) are triterpene saponins with multidirectional bioactivity. In this study, the contents of CE and ChIVa were determined in the roots, stems, leaves, and fruits of ten wild-growing species of Amaranthaceae. To achieve optimal extraction conditions for both saponins, maceration, shaking-assisted maceration, and ultrasound-assisted and heat reflux extraction were compared. A sensitive, specific, and rapid UPLC-MS/MS method was developed and validated for the simultaneous quantification of CE and ChIVa. The results showed that CE and ChIVa coexisted in most of the species analyzed, except for *Ch. hybridum*. For the first time, the presence of CE and ChIVa was noted in *L. polysperma*, *A. patula*, *B. bonus-henricus*, *O. rubra*, and *O. glauca*. Of the species analyzed, the highest ChIVa content was found in the fruit of *A. sagittata* (13.15 mg/g dw), *L. polysperma* (12.20 mg/g dw), and *Ch. album* (10.0 mg/g dw), and in the fruit and roots of *Ch. strictum* (5.52 and 7.77 mg/g dw, respectively). The highest amount of CE was determined in the fruit of *A. sagittata* (7.84 mg/g dw) and *Ch. strictum* (6.54 mg/g dw). These saponin-abundant plant parts of Amaranthaceae spp. may be considered convenient sources of these bioactive saponins.

## 1. Introduction

**Figure 1:**
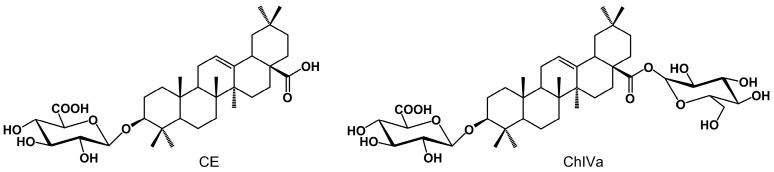
Structures of calenduloside E (CE) and chikusetsusaponin IVa (ChIVa).

Saponins are important secondary plant metabolites with an interesting bioactivity profile [1,2]. Calenduloside E (CE) and chikusetsusaponin IVa (ChIVa) (Figure 1) represent a group of pentacyclic triterpene saponins, with oleanolic acid (OA) as the aglycone and glucuronic acid (GlcA) attached to the C-3 position of OA. ChIVa, however, differs from CE in having an additional sugar moiety—glucose (Glc), ester-linked via a carboxyl group (28-COOH) at the C-17 position of OA.

Both OA-type saponins exhibit a wide range of biological activities, such as cytotoxic [3,4], anti-angiogenic, anti-tumor [3], and anti-hyaluronidase [5] properties. They also exert potent, well-documented anti-inflammatory effects via various mechanisms, including inhibition of NF-κB signaling and activation of the NLRP3 inflammasome. In addition, they inhibit pyroptosis by suppressing the PI3K/AKT/NF-κB signaling pathway and by triggering SIRT2 activity [6,7,8,9]. This has made them the subject of intense research into their potential use in the prevention and treatment of inflammation-related conditions, for example, obesity-related metabolic disorders [8], atherosclerosis [10], or rheumatoid arthritis [11].

Furthermore, CE also has cardioprotective effects in vitro [12,13] and attenuates myocardial ischemia/reperfusion (I/R) injury in animal models by suppressing calcium overload [14,15] and regulating mitochondrial fusion via the AMPK/optic atrophy 1 (OPA1) pathway [16]. This effect on mitochondrial function was found to be responsible for the neuroprotective role of CE during ischemic stroke [17]. Similar neuroprotective potential via the APN/AdipoR1/LKB1-mediated AMPK/GSK-3β pathway has also been reported for ChIVa in cerebral I/R injury in STZ-induced diabetic mice [18]. In addition, ChIVa could be beneficial in the prevention and treatment of type 2 diabetes, as it was demonstrated to exert hypoglycemic [19,20], anti-obesity [21], and protective effects on pancreatic β cells against intermittent high-glucose-induced damage and lipotoxicity [22,23]. Moreover, ChIVa was found to have in vitro antiviral properties, as well as antiherpetic efficacy against HSV-2 in a mouse model [24]. It was recently reported that ChIVa inhibits avian influenza virus (H9N2 AIV) replication in vitro and reduces virus-induced oxidative stress in vivo during H9N2 AIV infection [25]. Recent studies have shown that ChIVa can reduce ultraviolet-B (UVB)-induced skin photoaging [26] and acts as a lysine-specific demethylase 1 inhibitor to alleviate non-alcoholic fatty liver disease (NAFLD) induced by a high-fat diet [27].

Due to this remarkable, multi-target bioactive potential, both OA-type saponins, CE and ChIVa, have attracted considerable interest from the pharmaceutical industry. As a result, ongoing research is focusing on elucidating their mechanisms of action, enhancing their activity, and improving their bioavailability. For instance, some studies have explored the activity of synthetic CE analogs [12,28], while others focused on the bioavailability of CE and ChIVa in animal models (rats and beagle dogs) [29,30,31,32]. Pharmacokinetic studies have shown that both saponins have low bioavailability after oral administration [30,31,32]. However, Shi et al. [29] reported that the area under the plasma concentration–time curve (AUC_(0−t)_) for oral doses of CE is proportional to the dose administered. Recent studies have demonstrated that the main routes of oral absorption of ChIVa are the upper intestinal tracts [30], with some studies suggesting that ChIVa may undergo enterohepatic recirculation [31] as well as biotransformation by the intestinal microflora [33]. Furthermore, based on the research of Zhang et al. [30], ChIVa was classified in the Biopharmaceutics Classification System (BCS) as a substance with high solubility but low permeability (class III) after oral administration. A more recent study in animal models highlights the potential of ChIVa as a bioactive agent in liposomal formulations, capable of crossing the blood–brain barrier, for the treatment of acute ischemic stroke [34].

As significant amounts of these compounds are needed, plant sources of saponins are still being sought. CE is considered to be the predominant bioactive component in *Aralia elata* [29,35], while ChIVa is one of the saponin quality markers of *Panax japonicus* [36]. Phytochemical studies showed that both compounds were also detected in species belonging to the Amaranthaceae family, such as *Achyranthes bidentata*, *Bassia muricata*, *Chenopodium album* L. [37], *Salicornia bigelovii* [38], *Alternanthera philoxeroides* [24], and *Beta vulgaris* var. *vulgaris* [39]. Furthermore, in our previous work, we isolated these saponins from *Ch. strictum* [4] and *A. sagittata* [6].

Recently, some plants of the Amaranthaceae family, such as *Corispermum chinganicum* and *Achyranthes bidentata*, have become the subject of Chinese patents, covering the isolation method of CE [40,41]. Despite this fact, most published studies on CE and ChIVa have focused on the detection and isolation of these compounds from plant material, while existing data on their quantification in Amaranthaceae plants are scarce and limited to commercially cultivated plant species such as *Beta vulgaris* or *Achyranthes bidentata* [42,43,44,45]. Consequently, there is a gap in knowledge regarding the analysis of CE and ChIVa contents in various species of the Amaranthaceae family, especially those growing wild, and the distribution of these compounds in different plant parts.

Hence, the main objective of the present study was to determine and compare the contents of OA-type saponins (CE and ChIVa) in different morphological parts of ten species of Amaranthaceae growing wild in the natural habitats in Poland: *Amaranthus retroflexus* L.; *Atriplex patula* L.; *Atriplex sagittata* Borkh.; *Blitum bonus-henricus* (L.) Rchb.; *Chenopodiastrum hybridum* (L.) S.Fuentes, Uotila, and Borsch; *Chenopodium album* L.; *Chenopodium strictum* Roth; *Lipandra polysperma* (L.) S.Fuentes, Uotila, and Borsch; *Oxybasis glauca* (L.) S.Fuentes, Uotila, and Borsch; and *Oxybasis rubra* (L.) S.Fuentes, Uotila, and Borsch. Given that there are only a few reports on the concurrent quantification of CE and ChIVa in plant material [39,42,43,46], the secondary aim of this study was to develop and validate an UPLC-ESI-MS/MS method for the simultaneous determination of CE and ChIVa in the extracts, and to optimize the extraction procedure to achieve the best extraction efficiency of saponins from the plant material.

## 2. Results and Discussion

### 2.1. UPLC-MS/MS Analysis

LC-MS/MS techniques are currently widely used for the qualitative and quantitative analysis of plant metabolites. Triterpene saponins show poor absorbance in the short-wavelength range (below 210 nm), which often prevents their detection and reliable quantitative analysis in complex plant extracts using UV spectroscopic techniques. Since MS/MS identification does not depend on the presence of a chromophore in the molecule, it has become one of the most commonly used techniques in the analysis of triterpene saponins [47,48,49].

#### Method Validation

**Figure 2:**
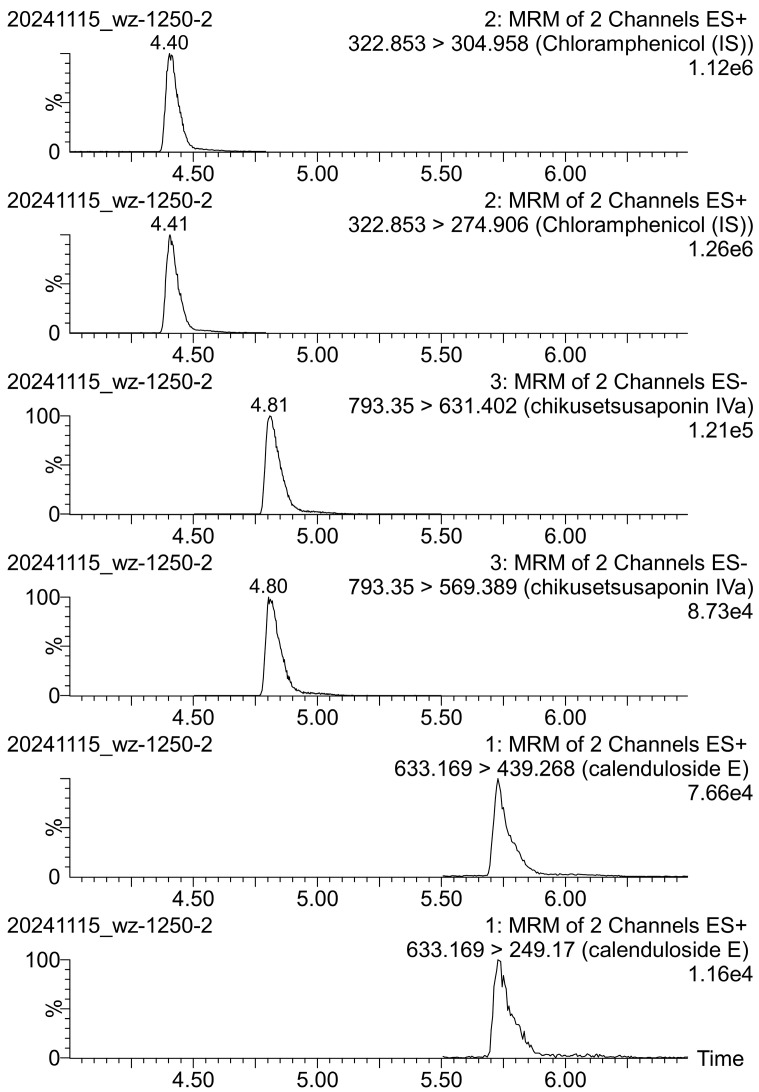
Multiple reaction monitoring (MRM) chromatograms of chloramphenicol (IS) and standard saponins: chikusetsusaponin IVa and calenduloside E.

In our previous work, we developed the UPLC-ESI-MS/MS method for the structural analysis of CE and ChIVa [4,5]. In the present work, to ensure the simultaneous determination of CE and ChIVa in the plant extract, and to achieve appropriate peak resolution, repeatability, and shortened analysis time, the conditions were adjusted to the most suitable parameters. As a result, the developed UPLC method was specific to CE and ChIVa, and it guaranteed obtaining well-shaped peaks. The method was found to be selective for the investigated compounds—the sum of the areas of peaks visible on the chromatograms of the blank solvent was lower than 5% of the AUC for samples at LOD concentration levels. Example MRM chromatograms for the analyzed saponins are shown in Figure 2.

Optimized settings for the quantitative analysis of both saponins and the internal standard (IS) are shown in Table 1. In the current study, chloramphenicol was chosen as the internal standard (IS) due to its similarity in retention time to the analyzed saponins under the analytical conditions employed, along with its good separation, signal intensity, and stability.

Based on regression analysis and Mandel’s fitting tests (*p* < 10^−6^ for both compounds), it was assumed that the calibration data fit the quadratic model well. The correlation coefficients and corrected determination coefficients (r^2^) obtained for the models were over 0.98. The distribution of the residuals can be well approximated with a normal distribution, as shown by the *p*-value of the normality test (Shapiro–Wilk)—*p* > 0.2.

The sensitivity of the method was good. The LOD and LOQ values for the saponins were below 240 and 310 ng/mL, respectively (Table 2).

Good precision and intermediate precision with % RSD less than 10% were observed. The ANOVA test showed no significant differences between analyses on different days (*p* > 0.4). Under all intentionally adjusted varied chromatographic conditions, including flow rate, column temperature, and mobile phase composition, the analyzed compounds were adequately resolved, and the elution order remained consistent. All of these results indicated the repeatability of the measurements. The matrix effect was found to be inhibitory and equaled −10.2% ± 0.4% and −14.5% ± 0.9% for ChIVa and CE, respectively. The obtained factors were used in further calculations. The results of the regression analysis are shown in Table 2.

Previously published studies that simultaneously determined CE and ChIVa contents in plant extracts were mainly based on HPLC-ELSD, HPLC-MS, UHPLC-MS/MS, and UPLC-PDA-HRMS methods [39,42,43,46]. The method developed by us was slightly faster (Rt of ChIVa ≈ 5 min and Rt of CE ≈ 6 min) compared to previously published procedures (Rt of ChIVa ≈ 5.06–48 min and Rt of CE ≈ 8.5–65 min) [39,42,43,46]. In addition, it also showed good sensitivity, higher than in most existing approaches for the simultaneous determination of ChIVa and CE [42,43]. However, it should be noted that the UHPLC-ESI-MS/MS procedure developed by Edelmann et al. [39] had a higher sensitivity for the determination of CE but lower sensitivity for ChIVa compared to our method.

### 2.2. Optimization of Extraction

The efficiency of saponin extraction from plant material is crucial to ensure their accurate and reliable quantification. Hence, prior to UPLC-MS/MS analysis, the extraction process was optimized using various techniques.

In numerous studies, the detection and quantitative analysis of saponins in extracts is preceded by extraction of the plant material using ethanol, methanol, or aqueous alcohol [50,51]. Reports on the extraction of CE and ChIVa from plant material provide data on the use of either aqueous alcohol (approximately 70% to 95%) [42,45,52,53] or pure alcohol (methanol or ethanol) [4,5,43,54]. In the current study, 80% methanol was used as an extractant, based on previous research on the simultaneous quantification of CE and ChIVa in plant extracts [42,43,46].

In this study, different morphological plant parts (roots, leaves, stems, and fruits) of ten Amaranthaceae species were analyzed (see Section 3.3, Materials and Methods). In only three of the analyzed species had the presence of CE and ChIVa been previously reported [4,5,55]. Hence, based on our previous studies, extraction optimization was carried out on *A. sagittata* fruit [5].

To select the most efficient procedure for the simultaneous extraction of CE and ChIVa from plant material, we compared different techniques commonly used in saponin extraction, such as maceration (ME), shaking-assisted maceration (ME/SA), heat reflux extraction (HRE), and sonication (UAE) [46,50,56,57]. To investigate the extraction efficiency of saponins from plant material [expressed as mg/g dw] depending on the amount of solvent used, the procedure were carried out in 1 to 4 cycles, using successive fresh portions of extractant in each cycle.

**Figure 3:**
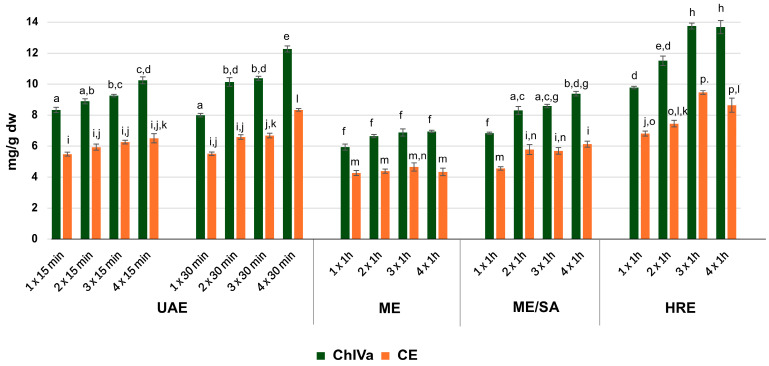
Average content [mg/g dry plant material] of calenduloside E (CE) and chikusetsusaponin IVa (ChIVa) in fruits of *A. sagittata*_S7 extracted by various methods: maceration (ME), shaking-assisted maceration (ME/SA), heat reflux extraction (HRE), and sonication (UAE). Mean values (n = 6) ± standard deviation (SD). Means with the same letter are not significantly different from each other (*p* > 0.05, F Welch’s ANOVA, followed by the Games–Howell post hoc test) within extracts analyzed for the content of ChIVa (a–h) and within extracts analyzed for the content of CE (i–p).

The results indicate that the use of different extraction techniques significantly affects the contents of saponins in the extracts (Figure 3). In the case of classical maceration (ME), the samples showed the lowest concentrations of CE and ChIVa, which suggests the need for longer exposure of the plant material to the solvent in order to extract saponins from within the plant. Some studies indicate up to 30-day (3 × 10 days) maceration, as demonstrated for saponins, including CE, from *Salicornia europaea* (Amaranthaceae) [52]. The results of our study suggest that the maceration time at room temperature was probably insufficient to obtain extracts rich in CE and ChIVa. In turn, the use of shaking-assisted maceration (ME/SA) improved the extraction efficiency of both CE and ChIVa compared to classical static ME (Figure 3). This clearly indicates that shaking ensures a more complete contact between the extractant and the plant material, thus increasing the diffusion of saponins from the plant tissue, as well as their dissolution in the solvent and, consequently, the extraction efficiency.

Numerous papers indicate that sonication is an effective technique, successfully applied to the analysis of triterpene saponins [48,58,59], including those found in Amaranthaceae plants [60]. Our study revealed that UAE allows for more efficient extraction of CE and ChIVa than both of the maceration methods tested. This suggests that the cavitation phenomenon, which ruptures the cell wall and releases compounds from the plant material [61], significantly enhances the extraction efficiency of saponins. For the ultrasound-assisted process, two extraction times were evaluated: 15 and 30 min per cycle, as the literature data suggest that a prolonged procedure may lead to compound degradation [58]. Extending the sonication time from 15 to 30 min slightly increases the saponin content in the extracts. However, statistically significant differences in saponin content were only observed in UAE extracts obtained in four consecutive cycles (4 × 30 min) using 50 mL of extractant per cycle. This method also yielded the highest levels of both saponins among all of the UAE processes analyzed. Although this procedure required more solvent, the saponin concentrations were lower than those obtained during a three-time extraction (3 × 1 h × 50 mL) of plant material using heat reflux extraction (HRE). The highest CE and ChIVa contents were obtained using the HRE technique (Figure 3), highlighting the importance of higher temperatures in improving extraction efficiency. The HRE and UAE methods are among the most used in saponin extraction [50,57]. Taking into account the same number of extraction cycles, our results indicating a higher extraction efficiency of saponins with the HRE method than with UAE are in agreement with previously published works [56,62,63].

On the other hand, considering the efficiency of the extraction process and its selectivity, it can be observed (Table 3) that despite the lower overall extraction yield (16.25%) obtained in the UAE extraction (4 × 30 min) compared to the HRE technique (3 × 1 h or 4 × 1 h) (18.51–19.28%), the saponin content in the extracts was approximately 7–7.5% and was comparable (Table 3). This indicates a higher selectivity of the sonication technique, allowing us to obtain CE-rich and ChIVa-rich extracts with lower ballast contents compared to HRE. Such extracts are important for isolating compounds and assessing the relationship between extract composition and pharmacological activity. However, in quantitative analysis, the percentage content of the analyzed substance in the extract does not always reflect its content in the plant material. Thus, it does not allow for a comparison of the substance contents in different plant materials or their quality.

In addition to the input of the extraction technique, the amount of extractant plays a crucial role in the efficient extraction of substances from plant materials. As shown in Figure 3, each successive extraction cycle using a fresh portion of solvent allowed the extract to reach a new equilibrium, thereby increasing the efficiency of saponin extraction from plant material during ME/S, HRE, and UAE. This trend was clearly visible for both saponins analyzed. Such an increase in saponin content, associated with successive extraction cycles, was also reported by other authors [58,62].

However, for the HRE technique, there were no statistically significant differences in the contents of both ChIVa and CE between the extracts prepared in three and four cycles. This suggests that a stable equilibrium was reached in the extract, so the amount of extractant used for the triple extraction was sufficient to extract both saponins by this method. In turn, the use of an adequate amount of solvent during a single 3 h extraction cycle (HRE 1 × 3 h) resulted in a statistically significantly lower saponin content (6.65 mg CE/g dw; 10.65 mg ChIVa/g dw) compared to extracts prepared by the HRE method (3 × 1 h). The results obtained confirmed that the addition of successive portions of solvent is crucial for extraction efficiency, and not only for the duration of the process.

Accordingly, heat reflux extraction carried out over three cycles demonstrated the highest extraction efficiency of triterpene saponins compared to the other procedures analyzed. Subsequently, extraction recovery tests were used to assess the accuracy of the HRE (3 × 1 h × 50 mL) process. The CE recovery was 98.58–102.39% (RSD = 1.89), and the ChIVa recovery was 97.25–101.37% (RSD = 2.20). In addition, no degradation of the analyzed saponins was observed during the procedure used.

In view of the results obtained, triple extraction by the HRE technique was chosen as an efficient method for the simultaneous quantification of ChIVa and CE in *A. sagittata* fruit. Since different analytical protocols make it impossible to compare results between the plant parts analyzed, this particular procedure, optimized for *A. sagittata* fruits, and providing efficient simultaneous extraction of CE and ChIVa even from plant materials with high contents of these saponins, was chosen to ensure the uniformity of procedures during studies comparing CE and ChIVa contents between the different plant parts of the ten Amaranthaceae species analyzed.

### 2.3. Quantitation of CE and ChIVa in Plants from the Amaranthaceae Family

In our search for rich sources of OA-type saponins among Amaranthaceae species, we focused on the plants growing in Poland that are relatively easily accessible in their natural habitat. We selected species in which CE and ChIVa have not been quantified so far. Furthermore, for some of the analyzed species, there are no reports whatsoever on the occurrence of CE and ChIVa. Triterpene saponins accumulate in different parts of plants [2]. In the Amaranthaceae family, CE and ChIVa have been reported in roots (*A. bidentata* [42,43], *Beta vulgaris* [45], *Ch. album* [55], *Ch. strictum* [4]) as well as aboveground parts (*A. nummularia* [64], *Salicornia bigelovii* [38], *S. europaea* [52]) such as leaves (*Beta vulgaris* [44]), flowers (*A. sagittata*) [5], and seeds (*Ch. quinoa*) [65]. Therefore, in our study, in order to determine the distribution of saponins in the morphological parts of the ten selected species, we analyzed their contents in different plant organs: roots, leaves, stems, and fruits.

Some data from the literature indicate that the contents of triterpene saponins in plant material may vary and depend on biotic and abiotic environmental factors or the vegetative stage of the plant [2,66,67,68]. Therefore, to investigate the possible variability in saponin concentrations, and to approximate the actual CE and ChIVa contents in the Amaranthaceae species studied, plant materials were collected at the fruiting phase from three different sites specific to each species, and at different harvest times (see Section 3.3, Materials and Methods).

Our study demonstrated that the saponin contents varied significantly both between species (in the same plant parts) and between different plant parts within individual species. The results obtained for plant materials from different locations are shown in Table 4.

Overall, our study revealed that CE and ChIVa were present in all analyzed species except for *Chenopodiastrum hybridum*, a species in which serjanic-acid-type but not oleanolic-acid-type saponins have so far been found [69]. Similar to *Atriplex* sp., *Chenopodium* sp., *Oxybasis* sp., and *Lipandra polysperma*, *Ch. hybridum* belongs to the tribe Atripliceae [70]; however, it was the only representative of this genus in the current study. For the sake of chemotaxonomic conclusions, future studies of CE and ChIVa contents should be extended to other representatives of the genus *Chenopodiastrum*.

The current quantitative study showed that ChIVa, unlike CE, was present in all morphological parts of the species in which the analyzed saponins were detected.

The ChIVa content in roots and fruits ranged from 0.05 to 9.30 and from 0.10 to 14.38 mg/g dw, respectively. The highest contents of this saponin were found in the fruits of *A. sagittata* (12.46–13.59 mg/g dw) and *L. polysperma* (9.66–14.38 mg/g dw). A slightly lower amount of ChIVa was determined in *Ch. album* fruit (9.46–10.82 mg/g dw), but this difference was not statistically significant. As for the fruit, only those from *Ch. strictum* and *A. sagittata* had high CE contents (5.09–8.11 and 7.26–8.17 mg/g dw, respectively).

Among the roots, the highest level of ChIVa was found in *Ch. strictum* (6.67–9.30 mg/g dw), followed by *Ch. album* (2.17–3.81 mg/g dw). Although the CE levels were lower than those of ChIVa, its highest concentration was also noted in these two *Chenopodium* species (1.66–3.90 mg/g dw). Referring to other plants of the Amaranthaceae family, the amounts of CE were higher than those determined for *Achyranthes bidentata* roots (0.51–2.7 mg/g dw), a medicinal plant material listed in the European Pharmacopoeia as well as the Pharmacopeia of China [42,43,71].

Our results show that although ChIVa dominated CE in most root samples, this trend was not apparent in the case of *A. retroflexus* and species of the genus *Oxybasis*. Similar observations for CE (18.5 mg/kg) and ChIVa were reported by Edelmann et al. in sugar beet roots (*Beta vulgaris* var. *vulgaris*) using the LC−MS/MS method [39]. In addition, the CE contents determined for the roots of most of the analyzed species were also higher than those reported by Mroczek et al. for the different sugar beet cultivars (1.4 μg/g dw to 0.48 mg/g dw) [44,45].

In contrast to the roots and fruits of the analyzed species of the Amaranthaceae family, the amount of each saponin in stems and leaves rarely exceeded 0.5 mg/g dw. The highest levels of both saponins in leaves were found in *A. sagittata*, while the highest levels in stems were found in *O. glauca*.

Our study showed that the quantitative profiles of ChIVa and CE in different Amaranthaceae plants varied. To investigate how closely the analyzed species are related to each other, and to observe whether there is a certain distribution pattern of the two saponins in individual morphological plant parts (irrespective of the place of origin of the species), a hierarchical cluster analysis was applied.

**Figure 4:**
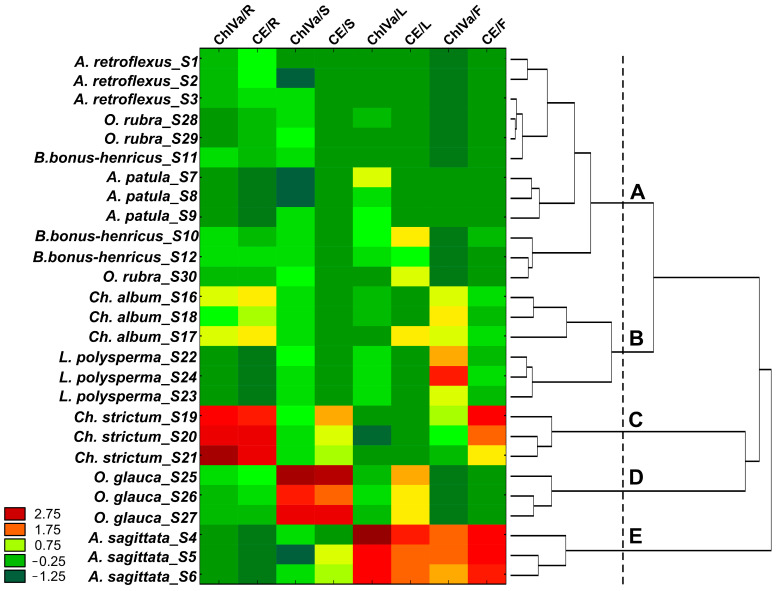
The heatmap and dendrogram to visualize the clustering of Amaranthaceae species based on the average contents (mg/g dw) of calenduloside E (CE) and chikusetsusaponin (ChIVa) in plant parts (R: roots, L: leaves, S: stems, F: fruits) of the species analyzed, determined for samples collected at different locations (method of grouping: Ward’s method). Clusters were marked with subsequent letters: A, B, C, D, and E.

The cluster analysis, based on the contents of CE and ChIVa in different morphological parts of Amaranthaceae plants, was performed using Ward’s method of grouping. According to Mojena’s rule and Grabiński’s measure, the species were divided into five main clusters: A, B, C, D, and E (Figure 4).

Cluster A was a multi-element group, comprising species characterized by stem CE contents below the LOQ or even the LOD. In these species, the concentrations of both saponins, regardless of the morphological part, were generally at relatively low levels. The greatest similarity was observed for *Blitum bonus-henricus* and *Oxybasis rubra*, in which the presence of both saponins was confirmed in all morphological parts. However, the CE concentration in the stems was below the LOQ.

The second group (Cluster B), containing two species (*Ch. album* and *L. polysperma*), similarly to Cluster A showed an absence or very low content of CE in the stems (<LOQ). Moreover, both species showed high concentrations of ChIVa in the fruits.

The remaining three clusters formed separate, single-element clusters (C, D, and E). In contrast to Clusters A and B, species classified into these groups contained quantifiable levels of CE in the stems (Figure 4).

Cluster C, containing only *Ch. strictum*, had high levels of both saponins (CE and ChIVa) in both fruits and roots, compared to other clusters.

The single-element Cluster D, comprising *O. glauca*, exhibited higher levels of both saponins in stems and roots than in fruits and leaves. It was characterized by a relatively high content of both saponins in the stems, which distinguished it from the other groups. The ChIVa content in the stems was three times higher (0.53–0.86 mg/g dw) than that determined in other species.

As for the last single-species cluster (Cluster E), containing *A. sagittata*, like Cluster C, it was characterized by high contents of both saponins (CE and ChIVa) in the fruit compared to other groups. However, in contrast to Cluster C, it also showed a low concentration of ChIVa in the roots, as well as a CE level below the LOQ in this morphological part.

The highest contents of ChIVa (up to 14.38 mg/g) and/or CE (up to 9.30 mg/g) saponins were recorded in species from Clusters B, C, and E. For most species in these groups, except for *L. polysperma*, there are previous data on the presence of CE and ChIVa in the roots or flowers [4,5]. However, to the best of our knowledge, in species from Clusters A and D, neither CE nor ChIVa has been reported so far.

The present work is the first comparative quantitative analysis of CE and ChIVa in selected species from the Amaranthaceae family, including analysis of individual morphological parts of the plants. In addition, our study allows for an assessment of the real value of the plant material as a source of CE and ChIVa saponins.

As shown in Table 4, despite some differences in CE and ChIVa concentrations in relation to the origin of the plant sample, there is a clear tendency for the analyzed saponins to accumulate in a specific part of the plant, characteristic of each species. For most of the representatives of the Amaranthaceae family, analyzed in the framework of this study, these were the fruits and roots. These observations are consistent with other studies on the accumulation of the triterpene saponin in other plants of the Amaranthaceae. Lim et al. showed that quinoa (*Chenopodium quinoa*) roots had the highest amount of total saponins (13.39 g/100 g), followed by quinoa bran (8.34 g/100 g) [72]. In contrast, a study on the aboveground parts of quinoa showed that although ChIVa was present in all parts analyzed, its highest amounts were found in the seeds and seed coats [65]. Another study, on sugar beet leaves and roots, revealed that ChIVa accumulated mainly in the leaves, while CE was only present in the roots [39]. Likewise, a study on beetroot cultivars showed a higher CE content in the roots (0.21–0.48 mg/g dw) than in the leaves (0.04–0.13 mg/g dw) of the plants [44].

It is worth noting that some of the Amaranthaceae species analyzed in our study had significantly higher CE and ChIVa contents (Table 4) than other representatives of this family analyzed to date, including *A. bidentata* (up to 0.51 mg CE/g dw of root and from 0.5 to 2.7 mg ChIVa/g dw of root) [39,42,43,44,45,71]. This is of interest because the roots of *A. bidentata*, as well as the aboveground parts of *Corispermum chinganicum* Iljin, are the subjects of patents on CE isolation, by which approximately 2.5 g of pure saponin can be obtained from 10 kg of plant material [40,41]. The natural habitat of these plants and others that are currently used to obtain CE (*Aralia elata*) and ChIVa (*Panax japonicus*) is Asia, where some of them are also cultivated. Our study has also shown that plant materials derived from European species of the Amaranthaceae family, such as the fruits of *A. sagittata*, *L. polysperma*, *Ch. album*, and *Ch. strictum*, as well as the roots of *Ch. strictum*, contain significant amounts of ChIVa (3.16–14.38 mg/g dw) as well as CE (5–9.30 mg/g dw), and should therefore be considered as potential sources of these saponins for commercial use.

## 3. Materials and Methods

### 3.1. Chemicals and Reagents

Methanol (analytical grade), LC/MS-grade acetonitrile, LC/MS-grade methanol, formic acid, and chloramphenicol (≥98%, TLC) were purchased from Sigma-Aldrich (St. Louis, MO, USA). HPLC-grade water was obtained from an HLP 5 (HYDROLAB Poland, Straszyn, Poland) apparatus and was filtered through a 0.2 µm filter before use. Chikusetsusaponin IVa (≥98%, HPLC) and calenduloside E (≥97%, HPLC) were isolated previously [4].

### 3.2. General Experimental Procedures

Ultrasound-assisted extraction was performed with the use of a Sonic-3 ultrasonic bath (POLSONIC, Warsaw, Poland). The Julabo SW20 shaker (Julabo Labortechnik GMBH, Seelbach, Germany) was used to perform shaking-assisted maceration. SPE extraction was performed using the Backer BAKER SPE 12G system on Backerbond SPE^TM^ C18 Polar Plus^®^ (1000 mg; 0.6 mL) cartridges (J.T. Backer, Phillipsburg, NJ, USA).

LC–MS/MS analysis was performed on a UPLC/MS Waters ACQUITY TQD (Waters Corporation, Milford, MA, USA) apparatus operated in the negative and positive electrospray ionization modes (see Section 3.11, Quantitative UPLC-MS/MS Analysis).

### 3.3. Plant Material

The plant material comprised 10 species of the Amaranthaceae family: *Amaranthus retroflexus* L.; *Atriplex patula* L.; *Atriplex sagittata* Borkh.; *Blitum bonus-henricus* (L.) Rchb.; *Chenopodiastrum hybridum* (L.) S.Fuentes, Uotila, and Borsch; *Chenopodium album* L.; *Chenopodium strictum* Roth; *Lipandra polysperma* (L.) S.Fuentes, Uotila, and Borsch; *Oxybasis glauca* (L.) S.Fuentes, Uotila, and Borsch; and *Oxybasis rubra* (L.) S.Fuentes, Uotila, and Borsch. Plant material was collected during the reproductive (fruiting) phase from July to September in 2019, 2020, 2023, and 2024. Each species was collected from three different locations in the southern part of Poland (Table 5). The collected plant species were identified by an expert botanist from the Department of Pharmacognosy, Jagiellonian University, Kraków, Poland. Voucher specimens (for reference numbers, see Table 5) were deposited at the Department of Pharmacognosy, Faculty of Pharmacy, Medical College, Jagiellonian University, Kraków, Poland.

The collected plant material was divided into leaves (L), stems (S), fruits (Fr), and roots (R) and then air-dried under controlled conditions (in the dark, at 24 °C, in an air-conditioned room) to a constant weight. The dried plant material was pulverized using a mechanical grinder (BOSCHMKM6003, BSHGmbH, Munich, Germany) and stored in airtight containers.

### 3.4. Selection of the Optimal Extraction Technique

The powdered plant material (fruits of *A. sagittata*_S4) was accurately weighed (1.0 g) and then extracted with 80% methanol (MeOH) using a variety of techniques: sonication (UE), maceration (ME), shaking-assisted maceration (ME/SE), and heat reflux extraction (HRE). For each extraction technique 1, 2, 3, and 4 extraction cycles were performed, each with a new portion of the extractant. Six extracts of plant material were prepared for each extraction procedure. The extractions were performed as follows:

Heat reflux extraction (HRE): Dried plant material was placed in a round-bottomed flask and extracted with 80% MeOH in a water bath (at temperature 80 °C) under reflux. The HRE procedures with the use of a plant material/solvent ratio (DSR) of 1:25 *w/v* were carried out as follows: 1 × for 1 h; 2 × for 1 h, 3 × for 1 h, 4 × for 1 h.

Extraction with 80% MeOH (plant material/solvent ratio (DSR) 1:150 *w*/*v*, 1 × for 3 h, in a water bath at 80 °C) under reflux was also performed.

Ultrasonic extraction (UE): A 1.0 g sample of plant material was placed in a conical flask and extracted for 15 min or 30 min with 50 mL of 80% methanol per extraction cycle. Number of extraction cycles tested: 1, 2, 3, and 4. Ultrasound-assisted extraction was performed at 25 °C with the use of a Sonic-3 ultrasonic bath.

Maceration (ME): A 1.0 g sample of plant material was placed in a conical flask and extracted with 80% methanol at room temperature (25 °C): 1 × 50 mL of solvent for 1 h; 2 × 50 mL of solvent for 1 h; 3 × 50 mL of solvent for 1 h; 4 × 50 mL of solvent for 1 h.

Shaking-assisted maceration (ME/SA): Extractions were performed in the same way as described for the maceration procedure (ME), but for dynamic maceration, the extracts were shaken using a laboratory Julabo SW20 shaker (JULABO GmbH, Seelbach, Germany, 25 °C; 100 U/min r.p.m.).

The extracts obtained were filtered using quantitative filter papers (POCH, Gliwice, Poland). In the case of multi-stage extraction, the extracts were combined. All plant extracts were then evaporated to dryness on a rotary evaporator and weighed to a constant mass.

### 3.5. Solid-Phase Extraction (SPE) Procedure

Solid-phase extraction was carried out based on conditions described previously [73], with some modifications. SPE C18 cartridges were used and conditioned with methanol, followed by water. The extracts were dissolved in 16 mL of water, and then 2 mL of sample was taken and applied to the cartridge. After loading the sample, the cartridge was washed successively with water (6 mL) and 20% methanol (12 mL). Finally, the saponins were eluted with methanol (30 mL). The resulting methanol fraction was evaporated under reduced pressure to a constant weight. Then MEOH SPE fraction was then redissolved in methanol and transferred to a 3 mL volumetric flask. Next, 1 mL from each fraction was transferred to vials and evaporated to dryness. The samples were stored in a refrigerator (5 °C) for further analysis.

### 3.6. Recovery Tests

Recovery tests were used to assess the accuracy of the method. Exact, defined amounts of reference saponins (CE and ChIVa) were added to 1.0 g of the sample (*A. sagittate* fruits, S7) or blank (solvent) and then extracted by the HRE method (3 × 1 h × 50 mL of 80% methanol) and analyzed. The recoveries [%] were calculated with the following formula:

Recovery (%) = (amount found − original amount)/amount spiked × 100%

RSD (%) = (SD/mean) ×100%

### 3.7. Optimized Extraction Procedure

Accurately weighed 1.0 g samples of plant parts (leaves, stems, fruits, and roots) from different species of the Amaranthaceae family were placed separately in round-bottomed flasks and extracted each with 50 mL of 80% methanol in a water bath (at 80 °C) under reflux. HRE was carried out for 1 h, and the process was repeated three times. The combined extracts were filtered (qualitative paper filters, POCH Gliwice), evaporated to dryness under reduced pressure, and weighed to a constant mass. Six extracts of each plant sample were prepared in the same way. The extracts were then subjected to the solid-phase extraction procedure as described in Section 3.5.

### 3.8. Preparation of Standards

An internal standard of chloramphenicol was weighed to 10 mg in a volumetric flask using an analytical balance. The volume was brought to 10 mL using methanol to give a solution of 1 mg/mL. Then, 1 mL of this solution was subsequently diluted to 10 mL with water in a volumetric flask to obtain a 100 μg/mL stock solution. This procedure was repeated to prepare a 10 μg/mL stock solution. Chikusetsusaponin IVa (ChIVa) and calenduloside E (CE) standards were weighed to 1000 μg in a volumetric flask using an analytical balance. The volume was brought to 1 mL using methanol to make stock solutions of 1000 μg/mL. These solutions were stored at −20 °C and used to make dilutions for the calibration curves.

### 3.9. Preparation of Calibration Samples

A dilution series of the 1000 μg/mL ChIVa and CE standard solutions was prepared by diluting 100 μL of both stock solutions with water, to a volume of 1 mL, and then diluting 500 μL of the resulting solution again with water, to a volume of 1 mL. This procedure was repeated several times to finally obtain a solution with a saponin concentration of 39 ng/mL. Then, 100 μL of the 10 μg/mL chloramphenicol internal standard solution was added to 500 μL of the dilutions of the CE and ChIVa standard solutions and diluted with water to a volume of 1 mL to obtain calibration samples with a chloramphenicol concentration of 1000 ng/mL and a concentration of the analyzed compounds in the range of 19.5–5000 ng/mL.

### 3.10. Preparation of Samples

From each sample, 100 μL of solution was taken, and 100 μL of 10 μg/mL chloramphenicol internal standard solution was added and brought to a volume of 1 mL with water, diluting the stock solutions of each sample. Each dilution was analyzed in triplicate by UPLC-MS/MS.

### 3.11. Quantitative UPLC-MS/MS Analysis

The UPLC-MS/MS system consisted of a Waters Acquity Premier (Waters Corporation, Milford, MA, USA) coupled with a Waters Xevo TQ-S mass spectrometer (electrospray ionization (ESI) mode–tandem quadrupole). Chromatographic separations were carried out using the Acquity UPLC BEH (bridged ethyl hybrid) C_18_ column (2.1 × 100 mm, and 1.7 µm particle size). The column was maintained at 40 °C and eluted under the following conditions: isocratic elution with 95% of eluent A over 1 min, followed by linear gradient elution from 95% to 0% of eluent A over 4 min and 100% of eluent B over 1.5 min, at a flow rate of 0.3 mL/min. Eluent A: water/formic acid (0.1%, *v*/*v*); eluent B: acetonitrile/formic acid (0.1%, *v*/*v*); 10 μL of each sample was injected in triplicate.

The Waters Xevo TQ-S mass spectrometer was calibrated for quantitative analysis using saponin solutions of 10 μg/mL at a flow rate of 20 μL/min and a 1:1 (*v*/*v*) mixture of eluents A and B at a flow rate of 0.28 mL/min. The optimized settings were as follows: source temperature 150 °C, desolvation temperature 250 °C, desolvation gas flow rate 600 L/h, cone gas flow rate 50 L/h, capillary potential 3.70 kV, collision gas flow 0.1 mL/min, and collision cell pressure 2.7 × 10^−3^ mBar. The cone potential and collision energy were optimized individually for each transition using saponin solutions (Table 1). Nitrogen was used for both the nebulizing and drying gases. Argon was used as the collision gas. Traces of the analyzed compounds were analyzed by the MRM (multiple reaction monitoring) method. A sum of both transitions was used for quantification. For confirmation of the identity of the analyzed compounds, peaks on both traces had to be visible.

### 3.12. Method Validation

The described UPLC-MS/MS method for the determination of ChIVa and CE was validated according to the ICH guidelines [74].

Specificity and selectivity: To demonstrate the specificity of the developed UHPLC-MS/MS method, the solutions containing the investigated compounds were analyzed. The selectivity was assessed by analyzing the blank solvent, along with standard solutions of the analyzed compounds at the LOD concentration level.

System suitability: Possible interferences between different compounds present in the analyzed samples were omitted by using the MRM method. The presence and identity of the compounds was verified by the presence of the peaks with appropriate retention times on both analyzed traces.

Linearity: The linearity of the investigated compounds was assessed by injecting eight separately prepared solutions covering the range of 19.5–5000 μg/mL of ChIVa and CE. During the statistical analysis, linear models and linearized nonlinear models (quadratic models) were analyzed:

Response = a_0_ + a_1_c (linear model), or

Response = a_0_ + a_1_c + a_2_c^2^ (qudratic model)

In the calculations, response was used, defined as response = AUC·c_int_/AUC_int_, where AUC is the area under the peak of the saponin, c_int_ is the concentration of the internal standard (ng/mL), and AUC_int_ is the area under the peak of the internal standard. For each compound, the sum of peaks on both traces was used in the calculations.

Limit of detection (LOD) and limit of quantification (LOQ): Based on the standard error of residuals (Se) and the slope (a) of the calibration plots, and following the definition of LOD and LOQ—i.e., LOD is the concentration estimated for the response equal to 10 Se, and LOQ is the concentration estimated for the response equal to 3.3 Se—the LOD and LOQ for the examined compounds were estimated.

Accuracy and precision: The accuracy and repeatability of the method were assessed by sixfold analysis of the concentration levels: 156 ng/mL (for ChIVa), 312 ng/mL (for CE), and 625 ng/mL and 5000 ng/mL for both of the saponins. The same protocol was followed for three subsequent different days to study the intermediate precision of the proposed method. The RSD (%) values of the peak areas of the saponins were calculated.

Robustness: To demonstrate the robustness of the method, deliberate small changes to the flow rate, acetonitrile content, and column temperature were made around the optimal values. The mobile phase flow rate was set to 0.30 mL/min. To evaluate its impact on resolution, the flow rate was changed to 0.27 and 0.33 mL/min. The effect of the column temperature was studied at 36 °C and 44 °C (instead of 40 °C), and the mobile phase composition was changed +5% from the initial composition.

Matrix effect: To assess the matrix effect of the method, five randomly selected extracts and five standard solutions in water at ChIVa and CE concentrations of 1000 ng/mL were analyzed. The same five extracts were spiked with 1000 ng/mL-ChIVa and CE and then analyzed, and the results were used in further calculations. Each analyzed sample contained the chloramphenicol internal standard at a concentration of 1000 ng/mL. The matrix effect was calculated according to the following equation:

Matrix effect [%] = [((Response_spiked_ − Response_unspiked_)/Response_standard_) − 1] × 100%

where Response_unspiked_ Response_spiked_, and Response_standard_ are the average response for the investigated compounds for the unspiked samples, spiked samples, and standard solutions in the solvent, respectively.

### 3.13. Data Analysis

All analytical data were processed using MassLynx V4.2 software (Waters Corporation, Milford, MA, USA). The statistical parameters of the calibration curves and quantitative data obtained during the analysis were calculated using the Statistica v. 13.3 program (TIBCO, StatSoft, Tulsa, OK, USA). The Shapiro–Wilk statistical test was used to determine whether the residuals and other data obtained had a normal distribution. Mandel’s fitting test was performed to check the linearity of the calibration curve. Levene’s test was used to check the homogeneity of variance. One-way analysis of variance (ANOVA) and Tukey’s post hoc multiple comparison test were used to test the statistical significance of inter-day differences. F Welch’s ANOVA and the post hoc Games–Howell test were used to determine the statistical significance of differences in saponin contents in the plant materials [mg/g dw]. The results were expressed as the mean (±SD). The probability level of *p* < 0.05 was considered statistically significant. Hierarchical cluster analysis (CA) (grouping: Ward’s method; function of the distance: Euclidean distance) was performed using Statistica v.13.3 (TIBCO Software Inc., Palo Alto, CA, USA). The chemical structures of the saponins were drawn using Signals ChemDraw v. 23.1.2.7 (Revvity Signals Software, Inc., Waltham, MA, USA). Graphs were generated in Excel 365 (Microsoft Office) or Statistica v. 13.3, while illustrations were prepared using CorelDraw 2021.5 (Corel Corporation, Ottawa, ON, Canada).

## 4. Conclusions

In conclusion, a specific and sensitive UPLC-MS/MS method was developed, validated, and successfully applied for the simultaneous quantification of CE and ChIVa in extracts from different morphological parts of ten Amaranthaceae species. The method demonstrated good chromatographic separation, was slightly faster, and showed higher sensitivity than most existing approaches for the concurrent determination of ChIVa and CE in plant samples. The developed UPLC-ESI-MS/MS method can be easily implemented in laboratories as a tool for the quantification of saponins in plant extracts and quality control of plant materials.

The results obtained in this study expand our knowledge about the representatives of the Amaranthaceae family and show that both analyzed saponins (CE and ChIVa) coexist in most of the analyzed species. To the best of our knowledge, this is the first report on the quantification of both bioactive saponins in the analyzed species. The presence and contents of CE and ChIVa were reported for the first time in the following species: *L. polysperma*, *A. patula*, *B. bonus henricus*, *O. rubra*, and *O. glauca.* Furthermore, no prior studies have reported the detection of saponins in *L. polysperma*, *A. patula*, and *O. glauca*. Given that saponins occur in plants as a mixture of compounds, this provides a basis for further research into these bioactive metabolites.

The findings of this study reveal significant differences in saponin accumulation both between the analyzed species and between specific morphological parts within a given species.

On the other hand, the current study did not detect the presence of any of the analyzed saponins in *Ch. hybridum*, which belongs to the genus *Chenopodiastrum*. These results indicate the need for further research to understand the factors affecting the synthesis and accumulation of saponins in plants of the Amaranthaceae family, and to extend the analyses to other species of the genus *Chenopodiastrum*.

In summary, the morphological parts of some plant species, including the fruits of *A. sagittata*, *L. polysperma*, and *Ch. album*, and the fruits and roots of *Ch. strictum*, are rich sources of ChIVa as well as CE. It should be noted that these are wild plants, often regarded as persistent weeds in cultivated fields. Therefore, when considering their use as a source of ChIVa and CE, the potential for their cultivation or alternative methods of obtaining them should be explored and evaluated.

## Figures and Tables

**Table 1:** Optimized settings for quantitative analysis of saponins and the internal standard.

Compound	Mode	Rt [min]	MRM Transition Q1→Q2 [*m*/*z*]	Cone Potential [V]	Collision Energy [eV]
Chikusetsusaponin IVa (ChIVa)	ES−	4.80	793.3→569.4	96	28
			793.3→631.4		20
Calenduloside E (CE)	ES−	5.73	633.2→249.2	16	24
			633.2→439.3		14
Chloramphenicol (IS)	ES+	4.40	323.1→275.0	16	10
			323.1→305.0		4

Q1—precursor ion/Q2—product ion.

**Table 2:** Regression analysis and method validation results.

Parameters	Chikusetsusaponin IVa	Calenduloside E
a_0_	−13.65 ± 0.97 (*p* < 10^−4^)	−9.27 ± 0.29 (*p* < 0.01)
a_1_	0.0987 ± 0.0049 (*p* < 10^−6^)	0.0432 ± 0.0047 (*p* < 10^−6^)
a_2_	−6.71 × 10^−6^ ± 0.99 × 10^−6^ (*p* < 10^−6^)	7.04 × 10^−6^ ± 0.96 × 10^−6^ (*p* < 10^−6^)
r	0.9947	0.9963
r^2^	0.9887	0.9920
LOD [ng/mL]	143	236
LOQ [ng/mL]	151	304
Calibration range [ng/mL]	151–5000	304–5000
Shapiro–Wilk test for residuals	*p* > 0.32	*p* > 0.21
Mandel’s fitting test	*p* < 10^−6^	*p* < 10^−6^
Asymmetry factor	2.3	3.1
Tailing factor	1.4	1.7
Concentration: 156 ng/mL:		
Accuracy [%]	105.1	nd
Intra-day RSD [%]	11.01	nd
Inter-day RSD [%]	12.60 (*p* > 0.21)	nd
Concentration: 312 ng/mL:		
Accuracy [%]	nd	92.8
Intra-day RSD [%]	nd	4.34
Inter-day RSD [%]	nd	6.24 (*p* > 0.67)
Concentration: 625 ng/mL:		
Accuracy [%]	94.2	91.8
Intra-day RSD [%]	7.88	8.32
Inter-day RSD [%]	9.02 (*p* > 0.42)	8.78 (*p* > 0.61)
Concentration: 5000 ng/mL:		
Accuracy [%]	98.3	99.4
Intra-day RSD [%]	1.38	0.13
Inter-day RSD [%]	5.21 (*p* > 0.52)	1.31 (*p* > 0.52)

nd—Not determined.

**Table 3:** Efficiency of various extraction procedures, expressed as extraction yield [%] and percentage content [%] of calenduloside E (CE) and chikusetsusaponin IVa (ChIVa) in the extracts from fruits of *A. sagittata*_S7.

Method of Extraction		Extraction Yield [%]	ChIVa Content [%]	CE Content [%]
UAE	1 × 15 min	12.11 ± 0.85	6.91 ± 0.50 ^a,b^	4.57 ± 0.31 ^a^
	2 × 15 min	14.76 ± 0.58	6.03 ± 0.29	3.97 ± 0.13
	3 × 15 min	16.05 ± 1.36	5.79 ± 0.43	3.94 ± 0.32
	4 × 15 min	15.98 ± 0.58	6.43 ± 0.36	4.29 ± 0.31
	1 × 30 min	12.96 ± 0.64	6.18 ± 0.34	4.33 ± 0.13 ^b^
	2 × 30 min	15.14 ± 0.40	6.71 ± 0.36 ^c^	4.25 ± 0.20
	3 × 30 min	15.37 ± 0.60	6.76 ± 0.26 ^d^	4.24 ± 0.18
	4 × 30 min	16.25 ± 0.76	7.56 ± 0.43 ^e,f,g,h^	5.13 ± 0.29 ^c,d,e,f,g^
ME	1 × 1 h	9.87 ± 2.03	6.22 ± 1.46	4.37 ± 1.12 ^h^
	2 × 1 h	12.51 ± 1.52	5.36 ± 0.61 ^e,i^	3.54 ± 0.43 ^c,i^
	3 × 1 h	13.51 ± 0.90	5.10 ± 0.17 ^a,f,j,k,l^	3.41 ± 0.05 ^d,j,k,l^
	4 × 1 h	14.26 ± 0.42	4.87 ± 0.05 ^b,c,d,g,m,n,o,p^	3.08 ± 0.03 ^a,b,e,h,m,n,o,p^
ME/SA	1 × 1 h	13.03 ± 1.69	5.30 ± 0.67 ^h,r^	3.60 ± 0.46 ^f,r^
	2 × 1 h	14.14 ± 1.53	5.94 ± 0.85	3.88 ± 0.67 ^g^
	3 × 1 h	14.26 ± 0.61	6.02 ± 0.08	4.02 ± 0.15
	4 × 1 h	15.07 ± 0.37	6.22 ± 0.10	4.15 ± 0.08
HRE	1 × 1 h	14.41 ± 1.10	6.81 ± 0.49 ^m^	4.70 ± 0.35 ^j,m^
	2 × 1 h	16.78 ± 1.80	6.92 ± 0.84 ^j,n^	4.55 ± 0.61 ^n^
	3 × 1 h	18.51 ± 1.02	7.44 ± 0.44 ^i,k,o,r^	5.10 ± 0.34 ^i,k,o,r^
	4 × 1 h	19.28 ± 0.81	7.11 ± 0.51 ^l,p^	4.72 ± 0.41 ^l,p^
	1 × 3 h	16.27 ± 0.42	6.55 ± 0.02	4.14 ± 0.06

Abbreviations: UAE—ultrasonic extraction, ME—maceration, ME/SA—shaking-assisted maceration, HRE—heat reflux extraction; Mean values (n = 6) ± standard deviation (SD). Means with the same letter are significantly different (*p* < 0.05, ANOVA, followed by Tukey’s multiple comparison test) within the columns.

**Table 4:** Average contents of chikusetsusaponin IVa (ChIVa) and calenduloside E (CE) in different plant parts of various Amaranthaceae species.

Species	ChIVa Content [mg/g dw]				CE Content [mg/g dw]			
	Roots	Stems	Leaves	Fruits	Roots	Stems	Leaves	Fruits
*A. retroflexus*_S1	0.57 ± 0.01	0.11 ± 0.08	0.16 ± 0.03	<LOQ	1.11 ± 0.04	<LOD	<LOQ	<LOD
*A. retroflexus*_S2	0.79 ± 0.03	<LOQ	0.16 ± 0.02	<LOQ	1.22 ± 0.03	<LOD	<LOQ	<LOD
*A. retroflexus*_S3	0.74 ± 0.02	0.17 ± 0.02	0.17 ± 0.04	<LOQ	0.80 ± 0.03	<LOQ	<LOD	<LOD
*A. sagittata*_S4	0.16 ± 0.02	0.17 ± 0.01	0.75 ± 0.05	13.59 ± 0.49	<LOQ	<LOQ	0.57 ± 0.12	8.17 ± 0.49
*A. sagittata*_S5	0.16 ± 0.01	<LOQ	0.56 ± 0.03	13.41 ± 0.86	<LOQ	0.35 ± 0.04	0.51 ± 0.02	8.08 ± 0.45
*A. sagittata*_S6	0.17 ± 0.02	0.17 ± 0.03	0.55 ± 0.09	12.46 ± 0.59	<LOQ	0.31 ± 0.08	0.48 ± 0.16	7.26 ± 0.64
*A. patula*_S7	0.19 ± 0.04	<LOQ	0.36 ± 0.08	1.11 ± 0.27	<LOQ	<LOD	<LOQ	0.41 ± 0.04
*A. patula*_S8	0.18 ± 0.03	<LOQ	0.24 ± 0.04	1.34 ± 0.09	<LOQ	<LOD	<LOQ	0.43 ± 0.02
*A. patula*_S9	0.19 ± 0.02	0.16 ± 0.03	0.3 ± 0.01	1.11 ± 0.32	<LOQ	<LOD	<LOQ	0.37 ± 0.05
*B. bonus-henricus*_S10	1.31 ± 0.06	0.18 ± 0.01	0.29 ± 0.01	0.21 ± 0.02	0.56 ± 0.04	<LOD	0.36 ± 0.05	0.57 ± 0.10
*B. bonus-henricus*_S11	1.33 ± 0.02	0.17 ± 0.05	0.17 ± 0.02	0.16 ± 0.01	0.57 ± 0.06	<LOD	<LOQ	0.41 ± 0.04
*B. bonus-henricus*_S12	1.35 ± 0.02	0.16 ± 0.03	0.23 ± 0.01	0.23 ± 0.01	0.83 ± 0.13	<LOQ	0.23 ± 0.11	0.48 ± 0.14
*Ch. hybridum*_S13	<LOD	<LOD	<LOD	<LOD	<LOD	<LOD	<LOD	<LOD
*Ch. hybridum*_S14	<LOD	<LOD	<LOD	<LOD	<LOD	<LOD	<LOD	<LOD
*Ch. hybridum*_S15	<LOD	<LOD	<LOD	<LOD	<LOD	<LOD	<LOD	<LOD
*Ch. album*_S16	3.68 ± 0.07	0.16 ± 0.01	0.19 ± 0.03	9.46 ± 0.29	2.41 ± 0.07	<LOQ	<LOQ	1.42 ± 0.15
*Ch. album*_S17	3.81 ± 0.3	0.19 ± 0.01	0.17 ± 0.01	9.73 ± 0.22	2.38 ± 0.03	<LOQ	0.4±	1.29 ± 0.19
*Ch. album*_S18	2.17 ± 0.04	0.18 ± 0.01	0.2 ± 0.01	10.82 ± 0.28	1.66 ± 0.02	<LOQ	<LOD	1.23 ± 0.23
*Ch. strictum*_S19	6.67 ± 0.09	0.21 ± 0.07	0.16 ± 0.02	8.12 ± 0.05	3.25 ± 0.45	0.47 ± 0.08	<LOQ	8.11 ± 0.06
*Ch. strictum*_S20	7.34 ± 0.08	0.19 ± 0.03	0.1 ± 0.04	5.3 ± 0.31	3.68 ± 0.04	0.34 ± 0.05	<LOQ	6.42 ± 0.07
*Ch. strictum*_S21	9.3 ± 0.17	0.19 ± 0.02	0.17 ± 0.08	3.16 ± 0.28	3.9 ± 0.53	0.32 ± 0.07	<LOQ	5.09 ± 0.14
*L. polysperma*_S22	0.17 ± 0.04	0.24 ± 0.04	0.25 ± 0.02	12.57 ± 0.64	<LOQ	<LOD	<LOQ	0.89 ± 0.04
*L. polysperma*_S23	0.17 ± 0.03	0.17 ± 0.01	0.25 ± 0.05	9.66 ± 1.09	<LOD	<LOD	<LOQ	0.54 ± 0.08
*L. polysperma*_S24	0.05 ± 0.01	0.16 ± 0.03	0.25 ± 0.07	14.38 ± 1.23	<LOD	<LOD	<LOQ	1.81 ± 0.24
*O. glauca*_S25	1.12 ± 0.04	0.68 ± 0.09	0.18 ± 0.03	<LOQ	1.03 ± 0.03	0.86 ± 0.13	0.43 ± 0.05	<LOD
*O. glauca*_S26	0.59 ± 0.03	0.48 ± 0.02	0.23 ± 0.01	<LOQ	0.79 ± 0.15	0.53 ± 0.09	0.39 ± 0.03	<LOD
*O. glauca*_S27	0.58 ± 0.09	0.55 ± 0.02	0.21 ± 0.02	0.1 ± 0.02	0.69 ± 0.09	0.73 ± 0.08	0.38 ± 0.03	<LOD
*O. rubra*_S28	0.33 ± 0.02	0.18 ± 0.03	0.19 ± 0.06	0.21 ± 0.08	0.71 ± 0.02	<LOQ	<LOQ	<LOQ
*O. rubra*_S29	0.4 ± 0.06	0.19 ± 0.04	0.17 ± 0.03	0.17 ± 0.03	0.69 ± 0.05	<LOQ	<LOQ	0.35 ± 0.02
*O. rubra*_S30	0.44 ± 0.07	0.2 ± 0.02	0.18 ± 0.07	0.18 ± 0.02	0.67 ± 0.03	<LOQ	0.32 ± 0.05	0.34 ± 0.06

Mean values (n = 6) ± standard deviation (SD); LOD—limit of detection; LOQ—limit of quantification.

**Table 5:** Data on analyzed plant material.

Species	Symbol	Collection Site: Geographical Coordinates	Date of Collection	Reference No. of Voucher Specimens
*Amaranthus retroflexus* L.	S1	49°55′05.6″ N 20°11′58.8″ E	July 2024	Fg/A.R/2024/07/1
	S2	50°02′19.4″ N 19°55′28.4″ E	August 2023	Fg/A.R/2023/08/2
	S3	50°01′50.3″ N 19°47′47.2″ E	August 2019	Fg/A.R/2019/08/2
*Atriplex patula* L.	S4	49°55′02.1″ N 20°12′02.1″ E	October 2024	Fg/A.P/2024/10/1
	S5	50°02′18.1″ N 19°55′26.2″ E	October 2024	Fg/A.P/2024/10/2
	S6	50°05′56.0″ N 19°54′24.1″ E	September 2020	Fg/A.P/2020/09/1
*Atriplex sagittata* Borkh. (syn. *Atriplex nitens* Schkuhr)	S7	50°21′29.1″ N 20°36′52.5″ E	September 2022	Fg/A.S/2022/09/1
	S8	50°02′50.7″ N 19°57′28.8″ E	August 2023	Fg/A.S/2023/08/1
	S9	50°02′27.5″ N 19°52′51.4″ E	August 2020	Fg/A.S/2020/08/1
*Blitum bonus-henricus* (L.) Rchb. (syn. *Chenopodium bonus-henricus* L.)	S10	50°00′44.0″ N 19°59′38.6″ E	August 2022	Fg/B.BH/2022/08/1
	S11	50°00′44.1″ N 19°59′40.2″ E	August 2020	Fg/B.BH/2020/08/1
	S12	49°49′26.2″ N 19°05′05.4″ E	September 2019	Fg/B.BH/2019/09/1
*Chenopodiastrum hybridum* (L.) S.Fuentes, Uotila, and Borsch (syn. *Chenopodium hybridum* L.)	S13	50°02′20.1″ N 19°55′26.3″ E	August 2021	Fg/Ch.H/2021/08/1
	S14	50°02′19.3″ N 19°55′28.1″ E	September 2024	Fg/Ch.H/2024/09/1
	S15	50°02′14.4″ N 19°49′18.9″ E	September 2023	Fg/Ch.H/2023/09/1
*Chenopodium album* L.	S16	49°54′53.1″ N 20°11′51.7″ E	August 2024	Fg/Ch.A/2024/08/4
	S17	49°56′54.7″ N 20°09′55.4″ E	August 2020	Fg/Ch.A/2020/08/1
	S18	50°02′21.7″ N 19°49′26.6″ E	August 2023	Fg/Ch.A/2023/08/2
*Chenopodium strictum* Roth (syn. *Ch. betaceum* Andrz.)	S19	49°54′53.1″ N 20°11′51.7″ E	August 2024	Fg/Ch.S/2024/08/1
	S20	50°00′42.7″ N 19°59′42.5″ E	July 2019	Fg/Ch.S/2019/07/2
	S21	50°00′43.2″ N 19°59′40.6″ E	August 2021	Fg/Ch.S/2021/08/1
*Lipandra polysperma* (L.) S.Fuentes, Uotila, and Borsch (syn. *Chenopodium polyspermum* L.)	S22	49°55′02.1″ N 20°12′02.1″ E	July 2024	Fg/L.P/2024/07/1
	S23	50°02′19.1″ N 19°55′27.5″ E	August 2023	Fg/L.P/2023/08/1
	S24	50°02′19.1″ N 19°55′27.5″ E	August 2022	Fg/L.P/2022/08/2
*Oxybasis glauca* (L.) S.Fuentes, Uotila, and Borsch (syn. *Chenopodium glaucum* L.)	S25	49°55′02.1″ N 20°12′02.1″ E	September 2024	Fg/O.G/2024/09/1
	S26	50°01′42.1″ N 19°47′49.9″ E	August 2023	Fg/O.G/2023/08/1
	S27	50°05′33.9″ N 19°54′43.9″ E	October 2020	Fg/O.G/2020/10/1
*Oxybasis rubra* (L.) S.Fuentes, Uotila, and Borsch (syn. *Chenopodium rubrum* L.)	S28	50°08′47.9″ N 20°34′34.4″ E	August 2017	Fg/O.R/2017/08/1
	S29	50°05′33.9″ N 19°54′43.9″ E	July 2022	Fg/O.R/2022/07/1
	S30	50°08′45.7″ N 20°34′36.9″ E	August 2020	Fg/O.R/2020/08/1

## Data Availability

Data are contained within the article.
